# Treating Emotion-Related Disorders in Japanese Traditional Medicine: Language, Patients and Doctors

**DOI:** 10.1007/s11013-012-9297-4

**Published:** 2012-12-30

**Authors:** Keiko Daidoji

**Affiliations:** Graduate School of Human Relations, Keio University, 4-1-1, Hiyoshi, Kohoku-ku, Yokohama, Kanagawa 223-0061 Japan

**Keywords:** Kampo medicine, Emotion-related disorders, Utsu, Neurasthenia, Constraint

## Abstract

This paper analyses how the conceptual and therapeutic formation of Japanese traditional medicine (Kampo) has been socially constructed through interactions with popular interpretations of illness. Taking the example of emotion-related disorders, this paper focuses on the changing meaning of constraint (utsu) in Kampo medicine. Utsu was once a name for one of the most frequently cited emotion-related disorders and pathological concerns during the Edo period. With the spread of Western medicine in the Meiji period, neurasthenia replaced utsu as the dominant emotion-related disorder in Japanese society. As a result, post-Meiji doctors developed other conceptual tools and strategies to respond to these new disease categories, innovations that continue to influence contemporary practitioners. I begin this history by focusing on Wada Tōkaku, a Japanese doctor of the Edo period who developed a unique theory and treatment strategy for utsu. Secondly, I examine. Yomuto Kyūshin and Mori Dōhaku, Kampo doctors of the early twentieth century, who privileged neurasthenia over utsu in their medical practice. The paper concludes with a discussion of the flexibility and complexity of Kampo medicine, how its theory and practices have been influenced by cross-cultural changes in medicine and society, while incorporating the popular experience of illness as well.

## Introduction

Traditional medicine tends to be associated with the image of a continuous and unchanging form of knowledge inherited from the past. However, many recent scholarly works, particularly with regards to the study of Chinese medicine, have challenged this assumption (e.g. Lucas 1982; Sivin 1988; Hsu 2001). In this paper, I will show that the same is true for the Japanese traditional medicine, commonly called Kampo 漢方.^1^ Kampo owes its theoretical and practical basis largely to not only the Chinese tradition, highly elaborated philosophical and natural studies of early China but also the innovative post-Song, late imperial innovations as well. Nonetheless, a historical account of Kampo formation shows that it was shaped by its clinical issues and the conditions unique to Japanese society to a far greater extent than is generally realized. Kampo has been moulded through a vernacularizing process that translated foreign ideas into local settings.

The elucidation of historical changes in medicine is an intricate procedure. The mechanism of medical knowledge is influenced by the complex interaction between social conditions, transnational influences, the economic and political climate, the scientific discoveries and so on. For instance, Fleck (1981) demonstrates the social construction of scientific knowledge by examining the relationship between the thought and experience of scientific facts. Through the historical account and epistemological consideration on the discovery of the Wasserman reaction, a test for syphilis, as a case study, the author demonstrates the interaction between the reformulations of the concept of venereal disease and socially and medically accepted criteria for diagnosis. This model of socially constructed medicine is convincing, but it lacks the voices of living people who are participants in the clinical scene. The studies of Kleinman (1980) on the understanding of illnesses as constructed through the relationship between healers and patients, based on the folk religious healing practices of Taiwan, direct our attention to this important perspective. He presents an innovative explanatory model which underscores the dialectical relationship between the social experience and cultural connotation of sufferings and the formation of health care systems and healing methods.

Taking advantage of these historical and medical anthropology methodologies, this article explores the mechanism of Kampo formation through the analysis of the social construction of medical knowledge and the patients’ experience of illness. Taking the case of emotion-related disorders, I will show in this paper that Kampo has undergone a complex process of transformation. Emotion-related disorders are closely related to the concept of utsu う つ in Japan. Nowadays, utsu is defined as depression, a growing public concern in Japanese society since the 1990s. With keen sensitivity to the process of translation when disease categories spread to new cultural settings (e.g. Young 1995; Cohen 1998; Kleinman 1986), medical anthropologists and historians have shed light on the complex formation of Japanese depression (Ohnuki-Tierney 1984; Lock 1987; Ozawa-de Silva 1996; Kitanaka 2012). But, utsu has a long history as a disease and pathology of emotion-related disorders in Japan. In the Edo period, this term meant ‘constraint 鬱’^2^ and was used to describe many of the emotional, psychological sufferings of these times. Constraint (utsu) indicates the undesirable state of ki 気 (qi in Chinese) within the body in which the smooth flow of ki is obstructed. Being one of the most essential and fundamental concepts in East Asian medical philosophy, the state of ki shapes all human conditions, both physical and mental. With the rapid spread of Western medicine in the late 19^th^ century, this utsu acquired new biomedical connotations, firstly in relation to nervous disorders and only much later to depression. Today, the Kampo usage of utsu and its biomedical meanings are in interaction with each other and continue to evolve.

Modern Kampo practitioners like to claim the effectiveness of Kampo treatments for depression, and other emotion-related conditions are based on the organic view of the body and mind in Kampo medicine. As the well-published Kampo doctor, Matsuhashi says: (e.g. Yamada 1979; Matsuhashi 1988)

Kampo medicine is characterized by treatments that view man as an organic and inseparable unity of mind and body… As the basic standpoint of psychiatry lies in dealing with a man as a whole, the perspective of Kampo medicine is advantageous to Western medicine which is liable to fall into the dualism of mind and body… (Matsuhashi 1988, p. 7).

Matsushashi’s views are widely shared among proponents of Kampo medicine, but they are in fact a particularly modernist perspective. By examining the history of emotion-related disorders, I will show that this ‘unity of body and mind’ has been debated and re-thought to a degree far greater than modern practitioners probably realize. In this paper, I will examine three representative doctors, Wada Tōkaku 和田東郭 (1744–1803) from the Edo period and Yumoto Kyūshin 湯本求真 (1876–1941) and Mori Dōhaku 森道伯 (1867–1931) from the post-Meiji period to trace these shifts in theory and practice. I will argue, as Kitanaka has also shown, that ki constraint was experienced widely as a category of disease in the Edo period (and as the manifestation of a culturally and socially depraved person) (Kitanaka 2004a, b; 2012). ‘In a peaceful age, there is no one who does not suffer from the constraint in the liver and gallbladder’.^3^ However, in the post-Meiji period, when neurotic theory came to Japan at the end of the nineteenth century, Kampo doctors abandoned the notion of ki constraint and adopted neurasthenia from their Western medicine counterparts as the primary lens for understanding emotion-related disorders (Watarai 2003; Kitanaka 2004a, b). In contrast to what Scheid and Karchmer have shown for the Chinese case (this issue), the impact of Western medicine in Japan had a profound effect on the direction of Kampo theory and practice in the early 20th century. By elucidating the complicated process of theorizing and treating emotion-related disorders between the 17th and 20th century, I hope to show how Kampo medicine has articulated with the power relations embedded in cross-cultural intellectual flows and adapted to changing understandings of emotion-related disorders in Japanese society.

## Intellectual Background of the Edo Period

Our story begins in the Edo period (1603–1867) when a long period of inner conflict ended and Kampo medicine entered upon a new phase of development. It was a dynamic period of innovation for Kampo when various medical theories and practices bloomed. Constraint was one interesting medical concept that doctors elaborated in new ways to explain professional and popular notions of emotional disorders. The richness of Edo period medicine was made possible by the Tokugawa Shogunate, which achieved military control over Japan, enacted an isolationist foreign policy, enforced a strict social hierarchy, and nurtured a growing monetary economy, manufacturing industry and flourishing cultural activities. Japanese medicine, which had previously rarely gone beyond the replication of trends in China also developed in a fluid intellectual space where various and unique perspectives arose. Because there was no collective body to authorize medical licenses and manage institutional medical education, many group and individual vagaries of medical fashion flourished. They presented various outlooks of medical theories and practices in order to accomplish their intellectual ambition as well as to meet the medical needs of the masses in the consumer society of the Edo period (Sakai 1982).

Our first protagonist, Wada Tōkaku 和田東郭 (1744–1803), like many physicians of this period was deeply influenced by the two dominant medical currents in pre-modern Japan. The first current was the Later Masters Currents 後世派 (Gosei-ha). Drawing upon the innovative post-Song medical reformers, the Later Masters Current emphasized the incorporation of neo-Confucianism into medical practice. Given this influence from a complex philosophical system, the medicine of the Later Masters Current also tended to be complicated and abstract. Historians have argued that the second current, the Ancient Formula Currents 古方派 (Kohō-ha), emerged from the criticism of the Later Masters Current. They believed that true medicine had been distorted by the misleading, neo-Confucian interpretations of later scholars and sought authenticity in clinical experience and the meticulous examination of the classic texts of ancient China. Among them, Yoshimasu Tōdō 吉益東洞 (1702–1773) was a unique physician of Ancient Formula Current. He rejected most of the foundational doctrines of China’s medical heritage as too abstract and speculative. Instead, he focused exclusively on the revered medical classic *Shanghanlun* 傷寒論 (*The Treatise on Cold Damage*) of Han China. Yoshimasu claimed that all treatments are for the purpose of attacking the pathogenic poison 毒 (doku) within the body, and that pathogenic poison is most palpable by abdominal diagnosis. As we shall see later, since Yoshimasu believed the human body to be isolated from the operation of heavenly ki and pathogens to localized and tangible objects, modern physicians often appreciated Yoshimasu’s approach as a pre-modern form of ‘empiricism’ (Ōtsuka 1996, pp. 27, 28).

These two currents, however, were unable to accommodate the vast exploratory spirit of Edo doctors, and other approaches also emerged. A small number of practitioners became interested in European medicine through the encounter with the Dutch, the only European power allowed to trade with Japan. They were known as doctors of Rampō 蘭方 (literally Dutch remedies). A large number of physicians, however, blended aspects of these various approaches, and historians have tended to categorize them as a fourth current, known as the Eclectic Current 折衷派 (Setchū-ha).

## Wada Tōkaku: All Diseases Arise from the Liver

Wada Tōkaku 和田東郭 (1744–1803) is typically considered part of the Eclectic Current in Kampo medicine. But, with respect to the development of the theory and treatment of emotion-related disorders, he is perhaps far more eclectic than most scholars have realized. He drew on popular notions of emotional illness and moral failings, combined them with uniquely Japanese debates about the role of the liver in medical theory, and reinterpreted the idea of constraint and gave it new and greater significance as a disease category. He is important for our story because he represents a highly articulated Japanese view of emotion-related disorders before the arrival of nervous theory from Western medicine. Wada was very successful in his time, becoming a court physician for the emperor in Kyoto. He was strongly influenced by Yoshimasu Tōdō, who was one of his medical teachers. As a result, Wada basically relied on abdominal diagnosis and the symptom-based approach of Ancient Formulas Current in diagnostic practice. But, in therapeutic practice, he also drew on the innovative post-Song doctors whose work forms the basis of the Later Masters Current.

Wada is best known for establishing a theory of pathology that ascribes all diseases to the liver. He classified all diseases into three types of liver disorders: the constraint of liver ki 肝気抑鬱 (kanki yoku’utsu), the agitation of liver fire 肝火聳動 (kanka shūdō) and the inherent liver poison 先天性の肝毒 (sentensei no kandoku). He observed that the constraint of ki in the liver and gallbladder was a widespread Japanese phenomenon during the peaceful reign of the Tokugawa regime:

We have been living in a peaceful era for a long time. This is why we commonly find all the people now have constraint in the liver and Gallbladder, and suffer from ki constraint (Wada 1821, p. 109).

Wada believed that the state of liver ki determines the state not only of one’s health but also of one’s personality. When there is too much spare time, one tends to be bothered with too much thinking or desire. Then, the liver ki loses its tension and ‘becomes blocked’ in the gallbladder. It results in the constraint of liver ki, producing excessive liver fire, which in turn can cause many disorders (Wada 1821, pp. 120–125). In this line of thinking, the state of one’s liver ki reveals how one has lived. Constraint as a category of disease and as a pathological mechanism was not only important to Wada but also to doctors of the Later Master’s School, who were inspired by the famous 14th century physician Zhu Danxi 朱丹渓 (1281–1358) from China. Zhu Danxi was known to these doctors as the first theorist of constraint, but Wada’s liver theory seems only be narrowly related to Zhu Danxi’s work. Rather, it is a highly contextualized articulation of medical theory with the popular experience of constraint. In the early Edo period, Japanese society experienced both rapid economic growth and political stability. In the mid-eighteenth century, however, economic development plateaued. A succession of severe famines on a national scale, widespread epidemics, numerous uprisings, and ongoing economic recessions, all gradually increased unrest and the sense of ‘stasis’ among the population (Yokota 2009; Yoshida 2009).

Wada’s understanding of ki constraint 気鬱 (ki-utsu) was strongly influenced by the increasing concerns about social stagnation and moral depravity among the general population. Historians and anthropologists have argued that the elaboration of constraint as a disease entity during this period was related to concerns about ‘how one lives one’s life’. For instance, Kuriyama (Kuriyama 1997) has argued that the association of ki stagnation with idleness was related to the flourishing of economy and industry during the Edo period, when the virtue of ‘being industrious’ was emphasized. The good circulation of ki is likened to the good circulation of capital, while the stagnation of ki was often considered a manifestation of the lack of hard work and the failure to nourish life. Kitanaka (2004a) has also pointed out that the pathology of ki constraint was basically considered to be at the root of emotion-related disorders during the Edo period. Recognized as the manifestation of pent-up emotions, from idleness to worry, frustration and resentment, ki constraint became a sign of moral failure. Rather than drawing on the notion of constraint as brought on by external (environmental) pathogens, as doctors from the Later Masters Current or many Chinese physicians might have at this time (see Scheid, this issue), Wada re-interpreted traditional ideas of desire and fire in shaping his notion of liver constraint.^4^

## The Liver and the Emotions

Wada’s understanding of the liver was also influenced by professional debates and popular confusion about three medical terms that presented translational challenges to doctors. Here, the significance of the oral dimension of the history of Japanese medicine should be underscored because phonetic sounds often played an important role in the development of Kampo. In pre-modern Japan, medical texts and terminologies mostly drew upon Chinese sources. However, there was (and still is) no systematic correspondence between Chinese ideographs and Japanese pronunciation. As Kuriyama (2004) has argued, the issue of translation in Japanese medical history not only brought a linguistic expansion to the Japanese language but also led to a conceptual fusion of new ideas. By imposing a Japanese reading on Chinese terms, ‘the result would in essence be a new word, resonating with the tension between the sense evoked in the Chinese character, and the meaning announced by the Japanese sound (Kuriyama 2004, p. 30)’. In other words, phonetic differences sometimes occurred when the gap between Chinese and Japanese pronunciation enlarged the difference in conceptual interpretation, and vice versa. Furthermore, it is often the case that colloquial readings were different from academic ones. It is therefore essential to know how terms were pronounced both in popular and medical literatures. In this sense, the Japanized sound for Chinese medical terms is a vector for the transmission of medical knowledge in two dimensions: from Chinese tradition to Japanese scholarly physicians and from Edo-period medical professionals to the ordinary Japanese commoners.

One of the keys to understanding the relevance of the liver to emotion-related disorders in Wada’s medicine, as well as Edo period medicine on the whole, is found in the three homonyms: kan 癇 (epilepsy), kan 疳 (tantrums) and kan 肝 (liver). Although they are not all pronounced the same in Chinese (*xián* 癇 and *gān* 疳, *gān* 肝), these three characters are pronounced the same in Japanese, *kan*. There was a debate and possibly some confusion about three ‘*kan*’ terms both in popular and medical languages (Azai 1865, pp. 327–330), epileptic *kan* 癇 came to refer to all kinds of twitching and convulsions. Tantrum *kan* 疳 was used to indicate many paediatric conditions, such as children’s fits of anger, crying at night or malnutrition. As the liver kan 肝 corresponds to anger in five phases theory, the liver symptoms were often associated with ‘bad temper’. As a result of confusion over the three terms, kan came to indicate not only all kinds of twitching or wrenching of the muscles, but often unusual personal characteristics and mental states as well. Even people who were pompous or obsessed with cleanliness were also regarded as afflicted with kan in everyday language (Kagawa 1788, pp. 383–385). Partly because of the same pronunciation and partly because of the cultural interpretation by which these three illnesses are often endowed with one’s mental or emotional states, they became confused. It firstly happened among laymen, some of who were not educated enough to strictly differentiate the meaning of each character. Although the nineteenth-century scholarly doctor, Azai Teian, saw this confusion as as ‘nothing but a mistake (Azai 1865, pp. 327–330)’, the muddled phrases of patients gradually began to affect some doctors.

These linguist ambiguities reinforced the centrality of the liver as the key to understanding emotion-related disorders in Wada’s medical practice. However, it is important to recognize the nexus between the liver and the emotions for Wada was very different than the views one finds in late imperial China. Wada was aware of some late imperial Chinese writings on the liver. For example, Wada commented approvingly on the work of Zhao Xianke, who was famous for saying that ‘All constraint is a liver disorder (Zhao et al. 1991, p. 50)’ and treating it with one master formula, rambling powder 逍遥散 (shōyōsan).^5^ But, Wada more commonly relied on formulas, such as dispersing the heart *Ki* drink 分心気飲 (bunshinki yin) or major seven-emotions decoction 大七気湯 (dai shichiki tō) of the Later Masters (Wada 1821, p. 147) that most physicians would not consider to be a liver treatment. Dispersing the heart Ki drink first appeared in the medical text *Heji jufang* 和剤局方 of the Song China (early 12th century) as treatment for all varieties of emotional disorders, in which congealed ki lodged in the chest or diaphragm. The majority of the herbs in this formula have acrid, dispersing, opening properties with an affinity for the Lungs, Spleen and Stomach. This formula has often been used for ki constraint since the Edo period (Ōtsuka 1963, 5th edn, 2000, pp. 247–248). Likewise, major seven-emotions decoction, uses similar herbs to treat emotional problems caused by ki constraint and phlegm obstruction.^6^ These prescriptions help clarify the unique role of the liver in Wada’s work. Wada described the liver as an ‘official’ or mediator, who managed all the inner organs and their treatments. For Wada, not only did all disease arise from the liver but also all treatments worked through the liver (Wada 1821, p. 151). This personification of the liver was also found in popular literature. For instance, one popular novel of the late eighteenth century compares the interior of a courtesan’s body to a merchant’s house, where the liver is the diligent butler who manages all the household affairs (Illustration 1).

**Illustration 1:**
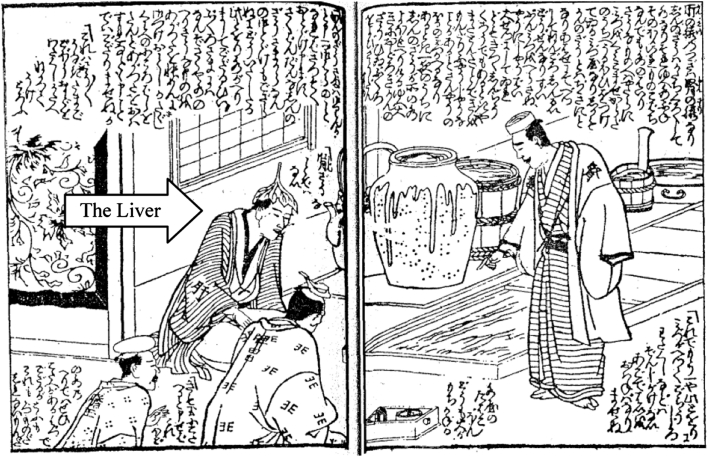
Fourteen courtesans inside the abdomen 十四傾城腹の内 (58–59) (*Fourteen courtesans inside the abdomen* (by Shiba 1793) is a parody of the acupuncture text *Japanese annotation of the fourteen vessels* 十四経絡発揮和解 by Okamoto Ippōshi 岡本一抱子, which is a commentary on *Annotation of fourteen vessels* 十四経発揮, a Chinese acupuncture text of the Yuan period by Hua Poren 滑伯仁)

## Talking Therapy for Liver Constraint

One of the unique features of Wada’s treatment for the liver constraint is ‘talking therapy 論説 (ronsetsu)’. But, unlike Freud’s talking cure or other variety of talking therapies in modern psychoanalysis, he emphasized conventional morality. This therapeutic practice reflected a melding of popular and professional views of emotion-related disorders of the times. Adapting the idea of morality into the pathology of constraint, Wada’s talking therapy shows that cultural connotations of chronic illness often lay beneath the expressiveness of disorders, into which doctor’s coping strategy is closely tied.^7^ Wada’s method included careful observation, followed by a revelation of the moral issues at the root of the patient’s problem. The following case is a typical example of Wada’s talking therapy for the treatment of liver constraint.

### The Case of Mrs. Mizuguchi 水口 in Kyoto (Female, Age 59)

Mrs. Mizuguchi, a widow of a head carpenter, had been in bed, unable to get up for 2 years. She had lost all of her eight children one by one. When her last surviving daughter died from an epidemic 2 years before, she had to adopt a son who was not a relation. Her relatives, friends and other doctors all sympathized with her situation. However, Wada perceived that she was just suffering from liver illness 肝疾 (kan-shitsu) that caused her constraint 鬱々 (utsu–utsu). He finally said to her:

Stop wailing and moaning, you fool and idiot!…The illness of ki constraint 気鬱 (ki-utsu) like yours never happens to those who have to earn their daily rice…If you were to ask me about the cause of your disease, I’d say it is nothing but smugness. You have completely forgotten that you are indebted to your ancestors (Wada 1821, pp. 170–179).

Convinced by Wada’s words, she apologized for her attitude and soon her illness was cured.

This case illustrates some of the typical characteristics of constraint illness in Edo-period Japan. Firstly, Mrs. Mizuguchi attributed her illness to physical ailments, believing the diagnosis by other doctors of ‘the emptiness and exhaustion of ki 気虚労役 (kikyo rōeki)’ which was usually presented as dizziness, sweating, palpitation and so on. Secondly, she was a social failure in the Edo-period context. Although she belonged to a relatively wealthy family, she failed to fulfil her expected role as a wife by giving birth to children and nurturing them to adulthood. The loss of eight children was not Mrs. Mizuguchi’s responsibility. However, the more ‘her grandchildren wept’, the greater the recognition of her social downfall. The patient blamed herself for her own social or familial failures, while her physical ailments prevented her from changing her unsatisfactory situation.

If we recall Kleinman’s notion of the explanatory model (1980, 1988), we might argue that Wada’s talking therapy tried to undermine the patient’s explanatory model by insisting that she not only suffered from an emotion related disorder but also a moral failure. The key concept in this re-interpretation was constraint (utsu). By expressing their states as ‘ki-utsu 気鬱’ or ‘utsu–utsu 鬱々’, Wada asserted that her suffering was not a serious disease. Instead of ki exhaustion or any other symptomatic diseases, Wada diagnosed her with constraint of liver ki due to moral depravity and an unproductive life. The importance of fulfilling one’s designated social duties fit well with the largely static social hierarchy set by the Tokugawa regime. Similarly, as Wada often reminded the patients of ‘the ancestors’ good deeds’, giving prominence to Confucianism and Buddhism teachings on the virtue of filial piety. Therefore, for those who felt their life aimless, the strategy of Wada’s talking therapy was to deliver the shock of a moral lesson by demonstrating the patient’s failure to be grateful to the ancestors and engage in hard work.

The case of Mrs. Mizuguchi illustrates the many dimensions of Wada’s approach to the treatment of the commonplace Edo period distress of constraint. Wada blended the multiple understandings of constraint, including its widely recognized moral causes, with the phonetic ambiguity surrounding the liver through pathology of fire and desire. This probably explains why Wada’s talking therapy, with its emphasis on the social duties and morality responsibilities of the patients, was often successful.

## From Constraint to Neurasthenia

The idea of constraint and the status of Kampo in Japanese society underwent a drastic shift in the late nineteenth century. The Tokugawa feudal government was overthrown by political reformers who revered the emperor, leading to the Meiji Restoration of 1868. The Meiji and post-Meiji governments were driven by a fear of Western imperialism and pushed Japanese society to embark on a rapid modernization program. The transition to modernity in Japan was so rapid and national in scale that many of the social customs and cultural ideas of the Edo period were rejected as ‘pre-modern’ or ‘old-fashioned’. It is therefore not surprising that the experience of emotion-related disorders in Japan also changed along with this historical transition from the pre-modern to modern period. Wada’s view of liver resonated with the middle to upper segments of society, which were most concerned about lack of vigour, in the stable, but static, Edo period. For those who had a sense of failure and a loss of aims, Wada’s treatment often awakened them to their neglect of family duties and work ethics. His unique understanding of the liver encompassed this complex pathology of constraint, mapping a new range of emotional disorders onto the basic framework of traditional medicine. In contrast, the Meiji Restoration (1868) brought the most rapid and drastic changes that the Japanese society had ever experienced. It is not surprising that constraint ‘utsu’, an illness of inactivity, soon did not conform to this quick moving society. When medical authorities began to use the term ‘utsu’ differently, Kampo doctors also found it necessary to explain emotion-related disorders through a different language and mechanism.

From the late nineteenth to the early twentieth century when the Japanese government rushed into modernization, neurasthenia became widely recognized as a new disease of the times. Instead of constraint, people began to express their depressed mood, symptoms of anxiety or fatigue with this imported disease category called shinkei-suijaku 神経衰弱 in Japanese.^8^ As the advertisement below suggests with its evocative heading, ‘the era of neurasthenia’ (Illustration 2), neurasthenia had replaced constraint illness as the most common form of everyday mental distress for Japanese people. The name of the drug itself, Leben (‘to live’ in German), hints at how widespread and disconcerting this apparently life-draining condition had already become in modern Japanese society. Neurasthenia was popularized by the American neurologist George Miller Beard (1839–1883) to describe the social experience of fatigue from rapid urbanization in the United States (Gijswijt-Hofstra and Porter 2001). The post-Meiji Japanese believed that the radical social transitions they were undergoing were also exhausting their nerves, and neurasthenia seemed to symbolize the mental and physical strains of their collective efforts to modernize Japanese society. (Watarai 2003; Kitanaka 2004a; Frühstück 2005).

**Illustration 2:**
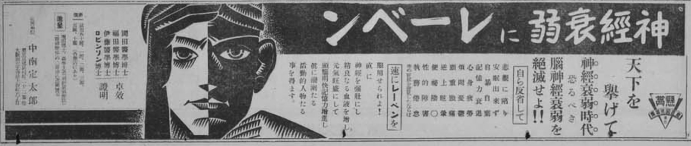
An advertisement for ‘Leben’, a medicine for neurasthenia, appearing in the Osaka-Asahi Newspaper, 29 June 1929, Osaka, Asahi-Shimbun-sha

The concept of constraint, particularly as illness category, lost its cultural and social basis when Western medicine was aggressively promoted in Meiji Japan. When the medical licensing Act was implemented in 1875 to institutionalize the educational standards, all examination subjects consisted of Western medicine. This system actually had a few loopholes that enabled most Kampo doctors to continue their medical practice.^9^ However, since anyone who wanted to become a qualified doctor thereafter had to master Western medicine, in the long run, Kampo’s future viability was endangered (Oberländer 1995; Sakai 1995; Suzuki 2007). Paralleling the diffusion of Western anatomical knowledge into Japan, constraint, like other concepts in Kampo, was re-interpreted and inscribed with a new meaning from Western medicine. Firstly, it was adapted to Galenic notions of emotional disorders. For instance, *Record of the correspondences between Chinese and Western disease names* 漢洋病名対照録 (Ochiai 1883, p. 99) glosses constraint as ‘melancholy (鬱憂)’ or ‘black bile disease (黒液病 or 膽液敗黒病 or 黒膽病).’ Subsequently, as melancholy and black bile disease transformed into brain disease in Western medicine, constraint soon became associated with brain diseases, in particular, the nervous disorders. A pioneer of modern psychiatry in Japan, Kure Shuzō 呉秀三 (1865–1932), re-defined utsu as a part of manic-depressive psychosis 躁鬱病 (sō-utsu byō) (Ishida et al. 1906). As a result, constraint as an independent diagnostic name basically disappeared from popular medical discourse, and according to Kitanaka (2004a), it was rarely found in the newspapers and magazines of the early twentieth century.

In the context of Kampo, however, constraint did not fade away, but its importance was diminished and its connotations shifted (Kitanaka 2004a). In the society where constraint was no longer a major complaint and Kampo was declining, some Kampo doctors tried to adapt medical theory to biomedicine disease categories, such as neurasthenia. They aimed to bring a ‘scientific outlook’ to Kampo medicine, not only to prove its compatibility with Western medicine but also to meet the changing complaints of patients. To illustrate these adaptive strategies, I will highlight two figures who exemplify the post-Meiji Kampo struggle for both professional and popular esteem: Yumoto Kyūshin 湯本求真 (1876–1941) of the Ancient Formula Current, Mori Dōhaku 森道伯 (1867–1931) of the Later Masters Current.

These doctors were part of the Kampo revival movement which made Kampo popular again in the 1930s. Shin (2011) points out that the leaders of this movement were Kampo doctors who had also learnt Western medicine. They promoted a reformed Kampo practice with the slogan ‘Kampo baptized by science’. Sensing new possibilities with the expansion of Japanese imperialism, these reformers encouraged Kampo doctors to go to China, Korea and Manchuria to modernize other East Asian traditional medical systems based on their innovations. Shin’s analysis elucidates how Japan’s imperial ambition and will for intellectual hegemony in East Asia became a driving force for medical reformation. The everyday demands of clinical work were another important factor. Since many Kampo reformers were certified in Western medicine in the first place, they used commonly neurasthenia, nervous disorders and other biomedical disease categories without reference to traditional ones. In addition, Kampo doctors had to show how their ideas and treatments had been ‘proven’ to get clients. Therefore, the reaction of Yumoto and Mori to the new biomedical category of neurasthenia will give us a useful insight into the conceptual and therapeutic transformation of Kampo which occurred in early twentieth century Japan.

### Yumoto Kyūshin: Neurasthenia as Autointoxication

In his treatments of neurasthenia, Yumoto Kyūshin turned to the Kampo idea of poison 毒 (doku). Yumoto was a proponent of the Ancient Formula Current, especially to the medicine of the Edo-period doctor, Yoshimasu Tōdō 吉益東洞. Arguing that Yoshimasu’s lack of speculative theory and adherence to abdominal palpation were precisely the sort of empiricism needed to reform declining Kampo medicine, Yumoto reconstructed Yoshimasu’s idea of poison. Yoshimasu claimed that there was one poison that was the cause of all diseases. Drawing on his knowledge of Western medicine, Yumoto claimed that this poison was the equivalent to ‘autointoxication’, a concept developed by Ilya Ilyich Mechnikov (1845–1916). In Yumoto’s *Sino*-*Japanese medicine* 皇漢医学 (1927), most cases of insomnia, neurasthenia, hysteria and neuralgia are attributed to urinary autointoxication 尿性自家中毒症 (nyōsei jika-chūdokushō), or in the language of Kampo medicine, water poison 水毒 (suidoku) due to malfunction of the kidneys (Yumoto 1927, vol. 1). In this explanation, we find a fusion of the new idea of Western toxin and the traditional idea of Kampo poison. In the early twentieth century, the Russian biologist Mechinikov developed a theory that ageing and all pathological malfunctions of the body were caused by the putrefaction of toxic bacteria in the gut, and the idea was soon introduced to Japan with a translation jika-chūdoku 自家中毒 (e.g. Tanaka 1909; Sugita 1933; Ishibashi 1935). Equating this type of poison with Yoshimasu’s definition, Yumoto asserted that all diseases were caused by autointoxication when the accumulated inner poison was not properly discharged due to the malfunction of the organs. In Yumoto’s usage, poison was not ascribed to bacteria, but to the malfunction of ki and blood within the body, thus implying that the new discovery of ‘autointoxication’ was already latent in the theory of a Japanese Kampo innovator from pre-modern times.

In contrast to Wada, Yumoto did not consider the social or cultural dimensions of neurasthenia in his treatment, instead focused entirely its somatic symptoms. He argued that emotion-related disorders, such as neurasthenia, hysteria, or hypochondria, all typically manifest as ‘fullness and pain in the chest and epigastrium 胸腹煩満 (kyōfuku han’man)’ or ‘stiffness and swelling in the lower abdomen 小腹鞭満 (shōfuku ben’man)’ (Yumoto 1927, vol. 1, pp. 38–39). Therefore, no matter what the diagnostic names are, prescriptions are to be determined by each patient’s constitution and his/her clinical presentation 証 (shō). For example, one man, aged over 50, was experiencing ‘constraint’ 鬱々 (utsu-utsu) and felt unhappy. He had shut himself up in a room for 3 years, frightened of a cock’s crowing or a dog’s barking, and often suffered from dizziness, uneasy sleep, nightmares and nocturnal emission. Palpating ‘fullness and pain in the chest and epigastrium’, Yumoto diagnosed this patient with neurasthenia, and prescribed two formulas^10^ to alleviate ki constraint [I may be changing your meaning here, but this works with the paragraph below]. These symptoms were cured in 3 months (Yumoto 1927, vol. 2, p. 64).

From the clinical case above, we observe that constraint, the term co-opted by Western doctors to mean nervous disorders, such as manic-depressive psychosis, was re-appropriated by Kampo again. In relation to neurasthenia, Yumoto used utsu mostly in order to describe the state of ki that generates poison within the body, in such terms as ki ‘stagnation 鬱滞 (utsu-tai)’ or ‘accumulation 鬱積 (utsu-seki)’. In other words, he did not recognise constraint as a diagnostic category any longer, but only as a pathological process (Yumoto 1927, vol. 1). Yumoto believed that poison alone did not cause a disorder, but constrained poison could manifest as a myriad of diseases, including neurasthenia. This notion of constrained poison was Yumoto’s attempt to formulate a Kampo explanation of the biomedical phenomenon of autointoxication.

## Mori Dōhaku: Three Constitutional Types and Neurasthenia

Mori Dōhaku 森道伯 also explained neurasthenia as the manifestation of inner poison due to ki constraint 気鬱 (ki utsu), but he traced his explanation to the 14th century Chinese physician, Zhu Danxi, reflecting his own medical lineage as the practitioner of the Later Masters Current. Mori eventually established his own unique brand of medicine called Ikkandō 一貫堂. He claimed that there are three constitutional types 三大証 to categorize Japanese people and that most diseases can be cured by just five formulas, if appropriately adjusted (Table 1). The names of the first two types, the stagnant blood constitution and the poisoned organ constitution, describe their main pathological tendencies; individuals of the third type, the detoxification constitution, tend to accumulate liver poison.

**Table 1:** Three constitutional types and five formulas of Mori Ikkandō medicine

The stagnant blood constitution 瘀血証体質 (Oketsu shō taishitsu)	Open and guide powder 通導散
The poisoned organ constitution 臓毒証体質 (Zōdoku shō taishitsu)	Saposhnikovia powder that sagely unblocks 防風通聖散
The detoxification constitution 解毒証体質 (Gedoku shō taishitsu)	Bupleurum decoction to clear the liver 柴胡清肝湯, Schizonepeta and forsythia decoction 荊芥連翹湯, Gentian decoction to drain the liver 龍胆瀉肝湯

Like all other diseases, Mori considered neurasthenia to be caused by the accumulation of inner poison. Unlike Yumoto, Mori argued that the manifestation of inner poison depends how it interacts with one’s constitutional type. There are three kinds of major poisons that cause internal disease: stagnant blood 瘀血 is the impure blood which mostly stagnates in the abdomen; food poison 食毒 is equated to Mechinikov’s autointoxication due to improper eating; water poison 水毒 refers to the excessively stagnant bodily fluids within the body due to the malfunction of the kidneys. When these inner poisons are induced by external causes (virus, bacteria, fatigue, etc.), it results in various disorders, both physical and mental. As the constitutional type mostly determines the nature and pattern of poison manifestation, finding out to which type the patient belongs is of prime importance. For instance, the stagnant blood constitution is likely to develop neurasthenia due to the rising of stagnant blood 上昇 to the head, therefore treatments should soothe and expel it (Yakazu 1964, p. 73). On the other hand, in the case of the Detoxification Constitution with neurasthenia, a different pathology is entailed and therefore treatment must be adjusted accordingly. Symptoms, such as insomnia, headache, tinnitus, palpitation, stiffness in the back and shoulders, loss of appetite and dullness in the limbs, are all evidence of liver poison. Therefore, the main treatment is to remove poison from the liver by schizonepeta and forsythia decoction, adjusted for the patient’s age and other personal conditions (Yakazu 1964, p. 149). Here, the connection between emotion-related disorders and the liver appears to resemble Wada’s, but Mori’s pathology is quite different and hence his treatment is too.

By claiming a causal relationship between constitutional type and neurasthenia, Mori’s pathology fit the generally accepted image of neurasthenia in this era as a constitutional or hereditary illness (Kitanaka 2004a). Although such theories as Hippocrates’s temperamental constitution and Kraepelin’s hereditary neurasthenia had been known in Japan, it was Morita Masatake 森田正馬 (1874–1938) who decisively linked neurasthenia to personal disposition. In the 1920s, he proposed a name shinkeishitsu 神経質 (literally nervous type) for those who have a tendency for neurasthenia, and established a unique psychological treatment, known as the Morita Therapy Method (Morita 1974, reprint 2004). Despite opposition by psychiatrists, this therapeutic method became popular, supported by numerous reports of successful cures. Mori’s neurasthenia lies midway between the disputes over the interpretation of neurasthenia, between the organic disease entity of Beard and Kraepelin and the personal disposition of Morita. Mori proposed that neurasthenia was ki constraint caused by the manifestation of inner poison in various forms, affected by one’s constitutional type and other external causes such as unhygienic life style. Therefore, the key to removing the poison, Mori claimed, is nothing, but improving one’s constitution and life style by proper eating habits, daily hygiene and appropriate herbal formulas when necessary. Morita Therapy, in contrast, dropped the need for herbal remedies altogether and extended the emphasis on correct lifestyle as the key to treatment.

### Poison: A Strategic Pathology

The accounts of Yumoto and Mori have two structural similarities, despite the fact that they claimed affiliation to different medical currents. One is the articulation of medical theory based on the concept of poison. The other is an almost exclusive focus on the clinical manifestation of the patient’s illness rather than on abstract theories of pathological mechanism. These two points suggest how post-Meiji doctors tried to adapt Kampo to contemporary needs while trying to preserve some of Kampo’s fundamental traits. In this process, constraint was also modified. The term retained its significance as a pathological concept, but its role as an illness category faded away.

The idea of poison has two advantages for the post-Meiji Kampo reformation. Firstly, poison was a bridge between the biomedical diagnosis and Kampo pathology. Trying to absorb the theory of autointoxication 自家中毒 (jika-chūdoku) into Kampo framework, both Yumoto and Mori took advantage the flexibility of the Kampo concept of poison. The equating of Western toxin to Kampo’s poison not only enabled these doctors to claim that this brand new biomedical discovery was already articulated by a Japanese doctor during the Edo period but also gave them grounds for claiming the superiority of Kampo over Western medicine: Western medicine was merely looking at the external causes of disease, such as bacteria, virus or physical fatigue in the case of neurasthenia. The real pathogen was the inner poison which creates the conditions for propagating a virus or any other external pathogens. Therefore, the true treatment is to restore bodily function by removing the poison from the body. In contrast to camphor injections to temporarily energize the neurasthenia patient or all kinds of serum treatment to kill external microbes (sometimes called a symptomatic treatment 対症療法, taisho ryōhō), Kampo could offer a more fundamental cure through its deeper insight into both internal and external disease causes. Such internal factors as one’s habits, constitution, medical history, personalities and gender, all mingled to determine the manifestation of poison (and lead to a causal treatment 原因療法, gen’in ryōhō). These polemics, which repeatedly appeared in the works of Yumoto and Mori, were a response to the criticism of medical officials who disparaged Kampo as mere symptomatic treatment (Fukagawa 1934, reprint 1981). Poison gave Kampo doctors that theoretical groundwork to claim professional status for their work.

Secondly, the idea of poison appealed to the popular imagination of illnesses in Japan. Throughout Japanese medical history, poison (doku) has been used to indicate numerous harmful and addictive materials and phenomena in everyday life as well as in medical work. For example, a widespread sexually transmitted disease, syphilis, was (and still is) called baidoku (literally plum-poison 梅毒 or mould-poison 黴毒), while the addiction to opium or drug is also expressed as chūdoku (struck by poison 中毒). In particular, opium addiction was a symbol of individual and national decay, as evidenced by China’s defeat in the Opium Wars (1840–1842), even in Japan. From food-poisoning (shoku chūdoku)^11^ to the belief that pimples and boils were the manifestation of inner poison (Endo 1981), the post-Meiji Japanese had long feared poison, both internal and external. Furthermore, the survey of the post-Meiji literatures shows that not only the physical body but also the mind is subject to be poisoned such as by literature, culture, ideology and science (e.g. 文学中毒, 文化中毒, 思想中毒, 科学万能主義中毒, etc.). These expressions suggest that ‘poison’ is both substantially and metaphorically pathogenic. Therefore, the strategy to associate Kampo poison with biomedical autointoxication was very catchy for the lay people who had long been familiar with poison aetiology. In other words, the idea of poison probably was often tied into the image of diseases and the fear of unhealthy dispositions among lay Japanese as well as medical professionals. It is perhaps not surprising that among these doctors, constraint became one type of poison, a toxic state of ki, blood and poison within the body.

### The Symptomatic Approach

By modifying the idea of poison and constraint, Yumoto and Mori introduced a new hybrid pathology to neurasthenia within the framework of Kampo. In treatment, their foci were either on somatic symptoms or constitutional type, and the biomedical diagnosis was actually of secondary importance. For example, while arguing that neurasthenia was caused by autointoxication, Yumoto emphasised that the key to treatment was not in finding the correct diagnostic name, but in how and where the inner poison manifested. Any one symptom might be shared across disease categories, such as ‘fullness and pain in the chest and epigastrium’ or ‘stiffness and swelling in the lower abdomen’, which is not only applicable to neurasthenia but also to amnesia, hysteria, myoma of the uterus, gastric cancer or oesophageal cancer and so on. But, the constellation of symptoms could reliably indicate the nature of the underlying poison and was therefore the most important step in prescribing proper formulas. On the other hand, in Mori’s medicine placed the prime importance on discerning the patient’s constitutional type at the outset of diagnosis. Constitutional types do not necessarily manifest alone, but they sometimes appear as a mixture in one person, therefore formulas should be customized carefully to suit each patient. Since symptoms are more or less predictable from constitutional type, they are given a mere secondary importance and diagnostic names even less importance. Consequently, the therapeutic methods of Yumoto and Mori suggest that in the post-Meiji Japan the Kampo’s medical reformations were a selective incorporation of Western medicine. Utilizing the diagnostic name of neurasthenia as a hinge, they partially adopted the elements of Western medicine to Kampo. Japanese reformists had to adapt to the new biomedical disease category, either through emphasizing the overlaps in two notions of poison or the differences between internal and external causes.

This makes a good contrast to the negotiation between traditional medicine and biomedicine in China. As Karchmer has argued, in Republican China, the integration of the traditional Chinese medicine with Western medicine was first formulated around specific understandings of the body. For instance, when the Republican medical reformists were trying to find a theoretical basis for the treatment of neurasthenia, they argued that the nerves were analogous to the liver, meaning that problems in nervous function could be addressed through liver therapies (see Karchmer in this issue). However, Yumoto and Mori did not perceive neurasthenia as directly affecting the liver. Yumoto claimed that neurasthenia was often caused by autointoxication which led to water poison in the kidneys. Although Mori felt that the liver detoxification constitution was the most likely to develop neurasthenia, he believed that many organs might be involved in the pathology of neurasthenia. This fact highlights the difference between medical reformation in China and Japan in the early twentieth century. Both Yumoto and Mori were skilful in creating Kampo pathologies for new biomedical disease entities, such as with the concept of poison. Unlike Chinese doctors of this era, they were not interested in rethinking Kampo’s basic physiology to make it consistent with biomedicine. As a result, the encounter with biomedicine led Kampo doctors to emphasize even more strongly the symptom-based approach, which was so distinctive in the Ancient Formula Current, but not limited exclusively to it, making it one of the most recognizable features of Kampo medicine today (Table 2).

**Table 2:** Transformation of emotion-related disorders in Kampo in two historical periods

	Patient’s complaint	Kampo explanation	Treatment	Resources
Edo-period				
Static	Constraint-utsu	Ki constraint	Formulas and talking therapy	Zhu Danxi, Ming-Qing Chinese medicine, treatise on the cold damages, Yoshimasu Tōdō, etc.
		Moral failure		
Post-Meiji period				
Changing	Neurasthenia	Manifestation of inner poison	The symptomatic approach	
		Constitutional type	Detox of the organs	

## Conclusion

To conclude, this paper has presented a historical account of the theoretical and therapeutic transformation of Kampo medicine through the treatment of emotion-related disorders. I have focused on the cases of constraint and neurasthenia, two of the major emotion-related disorders in the Edo period and the post-Meiji period. Affected by the specific socio-cultural influences in Japanese society and patient-doctor interactions, the mechanism of medical formation was a complex one. Wada, Yumoto and Mori each present a quite distinctive view of the emotion-related disorders that were widely experienced in their respective historical periods. In search of the explanations and treatments better suited to their times, these three doctors developed medical practices that were sensitive to changing social and cultural interpretation of illnesses.

Wada considered that liver constraint reflected the lack of vigour of those who were living in the peaceful, but static society of the Edo period. Seeing that liver constraint patients had a sense of failure and maladjustment to their society, Wada’s treatment often included the preaching of Confucian morality, such as family duty and work ethics. In his topology of the body, the liver integrated the social construct of constraint as an emotion-related disorder. Yumoto and Mori proposed a different understanding of emotion-related disorders. Because Western medicine had become the dominant form of medicine in Japanese society, neurasthenia was the representative illness of those suffering from emotional disorders. Attempting to equip Kampo with a ‘scientific’ outlook, these two doctors focused on pathogenic poison, equating it to biomedical autointoxication. This rhetoric not only enabled them to integrate the diagnosis of neurasthenia into Kampo medicine but also to utilise the image of pathogenic poison, which had been familiar to both lay people and doctors for so long. The response of Yumoto and Mori to Western medicine demonstrates that the shift between the Edo and post-Meiji periods was bigger and swifter than the changes that traditional medicine underwent in China during this period.

All three doctors were very sensitive to popular notions of emotional disorders. But, they embraced these ideas differently. Reflecting the fluidity of Edo period medicine, Wada was perhaps the freest and most eclectic of them all, borrowing from the major currents of medical thought as well as popular medical concerns to forge an original medical theory uniquely his own. Yumoto and Mori were more limited in their room for manoeuver. In order to prove the usefulness of Kampo in modernizing society, it was not possible for them to ignore new biomedical disease categories, such as neurasthenia. Unlike Chinese Republican medical reformers, they were not interested in re-thinking the fundamentals of Kampo physiology in relation to Western medicine. Although they creatively drew on resources within Kampo to develop new therapies, it is interesting to note that they emphasized only a few features of Kampo—poison, the symptom-based approach and so on—perhaps indicating the limits of their work in a world dominated by biomedicine.

Today, the meaning of utsu has changed once again to become the Japanese translation for depression, which was been the predominant type of emotion-related disorder in Japanese society since the 1980s. As contemporary Kampo doctors confront this new disease, they continue to demonstrate the theoretical flexibility and therapeutic efficacy of Kampo medicine by incorporating cross-cultural medical influences and popular conceptions of emotional problems into their practice.

## Footnotes

1 ‘Kampo’ was (and still is) by no means a monolithic establishment, without having central professional organizations. However in order to differentiate among the traditional medicines in China, Korea, and Japan, Kampo will be used to refer to Japanese traditional medicine in the reminder of this article.

2 In order to differentiate constraint-utsu from depression-utsu and from such Kampo pathological terms as seki 積 (accumulation) or tai 滞 (stagnation) that also involve obstructed ki state, I will use the translation ‘constraint’ for the utsu in the framework of Kampo.

3 Goto Konzan, *Notes of Master’s talk*, 1776, p. 10.

4 Huang (2011) claimed that the notions of desire and fire are closely associated with the pathology of constraint in Zhu Danxi’s medicine. However it seems more probable that Wada’s Liver constraint not only drew on Zhu Danxi’s desire and fire, but also on more eclectic sources.

5 For the detailed preparation and analysis of formulas, see Scheid et al. (2009), pp. 120–123.

6 See Scheid, Bensky, Ellis, Barolet (2009), p. 519.

7 Some medical historians consider Wada Tōkaku’s talking therapy as the forerunner of Morita therapy, a unique form of psychotherapy that appeared in early twentieth century Japan (Matsuda 2001, pp. 18, 19) or the pre-modern case of Narrative Based Medicine (Egashira 2007, p. 285). This modern evaluation, however, would need careful examination to prove such a direct link, and that is beyond the scope of this paper. As we will see below, talking therapy for Wada was very different than the sorts of therapies psychoanalysts later practiced in the 20th century.

8 神経衰弱 (shenjing shuairuo in Chinese) is used not only in Japan but also in China (Shapiro 1998, 2003).

9 Kampo doctors who had already practiced medicine before this Act were exempted from the examination. In 1882, this exemption was further extended to the point of granting the successors of Kampo practitioners with medical licenses without examination. Lock (1980) also pointed out that one of the reasons Kampo doctors could remain in post-Meiji was that Western medicine was too expensive and many doctors of Western medicine were not yet very well trained.

10 Yumoto prescribed bupleurum and cinnamon decoction 柴胡加桂湯 (to treat liver ki constraint and liver–stomach disharmony) and three coptis pill 三黄丸 (often to treat constipation). He sometimes added Purple Pill 紫圓 (to clear the stagnant poison in the chest and abdomen). See Scheid et al. (2009), pp. 109–110; Ōtsuka (2000), p. 372 and p. 762.

11 I have previously examined the idea of poison as a popular theme in the Edo-period dietary theory. Overeating does not just cause stagnation of foodstuff within the stomach, but the food itself rots and turns into poison. Therefore poison is not only traceable to the nature of foodstuffs, but potentially exists in every food, awaiting transformation into poison by a person’s improper diet and gluttony (Daidoji 2009).
